# Individuality in the Immune Repertoire and Induced Response of the Sponge *Halichondria panicea*

**DOI:** 10.3389/fimmu.2021.689051

**Published:** 2021-06-16

**Authors:** Lara Schmittmann, Sören Franzenburg, Lucía Pita

**Affiliations:** ^1^ Research Unit Marine Symbioses, GEOMAR Helmholtz Centre for Ocean Research Kiel, Kiel, Germany; ^2^ Research Group Genetics&Bioinformatics/Systems Immunology, Institute of Clinical Molecular Biology, Christian Albrechts University of Kiel, Kiel, Germany

**Keywords:** innate immunity, Porifera, LPS, host-microbe interaction, early-diverging metazoa, gene expression, RNA-seq, holobiont

## Abstract

The animal immune system mediates host-microbe interactions from the host perspective. Pattern recognition receptors (PRRs) and the downstream signaling cascades they induce are a central part of animal innate immunity. These molecular immune mechanisms are still not fully understood, particularly in terms of baseline immunity vs induced specific responses regulated upon microbial signals. Early-divergent phyla like sponges (Porifera) can help to identify the evolutionarily conserved mechanisms of immune signaling. We characterized both the expressed immune gene repertoire and the induced response to lipopolysaccharides (LPS) in *Halichondria panicea*, a promising model for sponge symbioses. We exposed sponges under controlled experimental conditions to bacterial LPS and performed RNA-seq on samples taken 1h and 6h after exposure. *H. panicea* possesses a diverse array of putative PRRs. While part of those PRRs was constitutively expressed in all analyzed sponges, the majority was expressed individual-specific and regardless of LPS treatment or timepoint. The induced immune response by LPS involved differential regulation of genes related to signaling and recognition, more specifically GTPases and post-translational regulation mechanisms like ubiquitination and phosphorylation. We have discovered individuality in both the immune receptor repertoire and the response to LPS, which may translate into holobiont fitness and susceptibility to stress. The three different layers of immune gene control observed in this study, - namely constitutive expression, individual-specific expression, and induced genes -, draw a complex picture of the innate immune gene regulation in *H. panicea*. Most likely this reflects synergistic interactions among the different components of immunity in their role to control and respond to a stable microbiome, seawater bacteria, and potential pathogens.

## Introduction

The core function of immunity is shared across animals: to differentiate between self and non-self, to maintain homeostasis, and to interact with microbes (1, 2). Immunity accompanied the evolution of multicellularity in response to the coexistence with microbial life, which already dominated our planet when animals emerged (3). Innate immunity is the most ancient and universal mechanism for host-microbe interactions and, even if vertebrates evolved adaptive immunity, they also strongly rely on their innate immunity (4). A key component of innate immunity is a variety of pattern-recognition receptors (PRRs), which detect microbes *via* conserved microbial-associated molecular patterns (MAMPs) like lipopolysaccharides (LPS), peptidoglycan, or flagellin (5). Among the most studied PRRs are: TLRs (Toll-like receptors), NLRs (nucleotide binding and leucine-rich repeat receptors), CTLD genes (C-type lectin like domain genes), and SRCRs (scavenger receptor cysteine-rich). In addition to classical PRRs, other receptor classes can detect microbial signals, among them GPCRs (G protein-coupled receptors) and cytokine receptors (6, 7). Some PRR families are highly diversified in invertebrates suggesting their potential for specific recognition (reviewed in 8). Traditionally, the evolution of PRR diversity has been seen as an “arms race” against pathogens (4, 9). But since recent, evidence suggests that PRRs also detect commensal microbes, promoting homeostasis (reviewed in 10).

The signals detected by PRRs are amplified *via* signaling cascades in order that the corresponding immune response can be mounted. Upon MAMP binding, induced transcriptomic responses can either intensify or also dampen the immune response, in a context-dependent manner (11, 12). It remains largely unknown which transcriptional mechanisms of signal transduction respond to different MAMPs, how they determine the specific response to pathogens or commensals, and how they might differ between and within animal phyla. On the one hand, the genetically available immune repertoire will determine the potential response of an animal. On the other hand, the realized (expressed) immune repertoire often differs from the potential repertoire and the expressed genes prior to microbe encounter are relevant to the response that is mounted (13). It is thus important to characterize the molecular components of the baseline immunity and the induced responses to get a comprehensive picture of the mechanisms mediating animal-microbe interactions.

Sponges (phylum Porifera) as early-diverging metazoans provide information about the origin and early evolution of innate immunity. They harbor a specific and stable microbiome (14) while feeding on microbes from the seawater (15, 16). Intriguingly, only one opportunistic sponge pathogen has been discovered so far (17). The first sponge genome, that of the Great Barrier reef sponge *Amphimedon queenslandica* (18), revealed a complex repertoire of immune receptors, including NLRs, SRCRs, and non-canonical TLRs (19). Canonical TLRs are comprised of an intracellular TIR domain and extracellular LRRs (leucine-rich repeats), but sponge TLR-like receptors consist of the TIR domain [homologous to the TIR domain in vertebrate TLRs (20)], combined with extracellular immunoglobulin domains (19). Importantly, the PRR families NLR, GPCR, and SRCR are diversified in *A. queenslandica* (21–23) suggesting their potential for microbial differentiation (24). The genomes and transcriptomes generated so far confirmed that the diverse repertoire of PRRs and presence of TLR- mediated signaling cascades occur in other sponge species, too (20, 25, 26). However, the diversification of certain families and the induced response upon MAMP challenge may as well depend on the microbial density associated with sponges (26, 27). Based on the microbial density, sponges are classified as either high or low microbial abundance sponges (HMA or LMA, respectively) (28, 29). LMA sponges harbor two to four orders of magnitude less bacteria than HMA sponges (30). Still, the field of sponge immunity is at its infancy. It is largely unclear how sponges recognize and respond to bacteria and whether those mechanisms are conserved across this phylum, are linked to the HMA-LMA dichotomy, or are rather species-specific.

We aim to characterize the expressed immune repertoire and the induced response in the breadcrumb sponge *Halichondria panicea*, a promising model for sponge symbioses (31), by ways of RNA-seq. We explored both the repertoire and gene expression patterns of PRRs, as well as the induced immune response to bacterial LPS. *H. panicea* is an LMA sponge and is dominated by an extracellular alphaproteobacterial symbiont that is unique to this sponge species (32, 33). Our results provide a first understanding on the innate immune system of *H. panicea* in the context of sponge-microbe interactions.

## Materials and Methods

### Sponge Collection and LPS Challenge

Twelve individuals of the breadcrumb sponge *H. panicea* were collected close to the shore at ~2 m depth in Kiel, Germany (54.424278, 10.175794) on 10.07.2018 and directly transferred to an open flow-through aquarium system at KIMMOCC facilities in GEOMAR Helmholtz Centre for Ocean Research, Kiel, Germany. We defined sponge individuals as these were collected from the same location but from distinct rocky crevices. Four weeks prior to the experiment, sponges were transferred to a closed re-circulation aquarium system with a mechanical and biological filter unit and thus reduced bacterial load. Each sponge individual was placed in separate 12 L aquariums and divided into 2 equally sized clones (~2x2x3 cm). In the closed system, sponges were fed five times a week with powdered *Nannochloropsis salina* algae in sterile filtered saltwater (~ 6000 cells/mL, Algova, Germany). Water was constantly mixed by pressurized air supplied through 2 mL serological glass pipets. For the duration of the experiment on the 25.09.2018, the recirculation was stopped and experiments were performed at 15.1°C and a salinity of 15.4 PSU. The treatment was started by either injecting sponges with 500 µL of LPS at a concentration of 1 mg/mL (Escherichia coli O55:B5, Sigma L2880) in filtered sterile artificial seawater (LPS treatment), or with 500 µL filtered sterile artificial seawater as sham control (ASW control treatment). LPS or ASW were injected by piercing the sponges at 5 different locations with a syringe and needle (diameter 0.45 mm) and injecting 100 µL each time. This way, the treatment was distributed through the tissue and a local response prevented. Samples were taken 1 h and 6 h after treatment. Importantly, the same sponge individual was sampled at both time points (clones of the same individual), but not from both treatments (different individuals per treatment). The sponge tissue samples were cleaned from algae and rinsed with sterile filtered ASW before preservation in RNAlater. Samples were first stored at 4°C overnight and subsequently frozen at -80°C until RNA extraction. This experimental design consisted of 2 treatments x 2 time points x 6 replicates (5 replicates in the LPS treatment at 6 h due to poor RNA quality).

### Eukaryotic Total RNA Extraction and Sequencing

Eukaryotic total RNA was extracted from ~70-80 mg tissue with the AllPrep DNA/RNA Mini Kit (Qiagen, Netherlands) according to the manufactures’ protocol. Degradation of RNA was inhibited by application of SUPERase-IN (Thermo Fisher Scientific, USA) at 1 U/µL and genomic DNA was removed post extraction (DNA-free DNA removal Kit, Thermo Fisher Scientific, USA). Successful removal of prokaryotic and eukaryotic DNA was verified by PCR and gel electrophoresis [18S rRNA primer Sp18aF 5’CCTGCCAGTAGTCATATGCTT, Sp18gR 5’CCTTGTTACGACTTTTACTTCCT (34), 16S rRNA gene primers Eco8F 5’AGAGTTTGATCCTGGCTCAG, 1492R 5’GGTTACCTTGTTACGACTT (35)]. RNA was quantified in Qubit (RNA BR Kit, Thermo Fisher Scientific, USA) and its quality checked spectrophotometrically (NanoDrop, Thermo Scientific, USA) and with automated electrophoresis (Experion, Bio-Rad, USA). RNA extracts were normalized to 50 ng/µL per sample by dilution with the Qiagen elution buffer. Library preparation (TruSeq stranded mRNA kit with poly-A enrichment, Illumina, USA) and paired-end sequencing (NovaSeq S1 2x150 bp, Illumina, USA) were performed at the IKMB Kiel, Germany.

### *De Novo* Transcriptome Assembly and Annotation

Adapter trimming and quality filtering of the raw sequencing reads was performed with Trimmomatic (36) (version 0.35, parameters LEADING:3 TRAILING:3 MINLEN:120). The read quality was manually checked in FastQC (version 0.11.8). Prokaryotic and microbial eukaryotic reads were removed with Kaiju (37) (version 1.7.2) in greedy-5 mode. Due to the lack of a reference genome for *H. panicea*, *de novo* transcriptome assembly was performed in Trinity (38) (version 2.8.5). The assembly was analyzed for completeness by comparing the longest isoforms of each Trinity component to the metazoan reference database for conserved genes with the BUSCO approach (39) (version 3.0.1). Annotation was performed with Trinotate (40) (version 3.2.0) by comparison to publicly available data (Blast+, SwissProt), protein domain identification (HMMER, Pfam), protein signal peptide and transmembrane domain prediction (signalP, tmHMM), as well as eggnog, GO and KEGG annotation. Contigs matching bacteria, archaea and viruses (based on blast results) were removed. The transcripts and the translated coding regions predicted by TransDecoder as part of the Trinotate pipeline (> 100 amino acids) were compared to the proteome of the sponge *A. queenslandica* (Uniprot UP000007879_444682) by blastx and blastp, respectively (e-value < 1e-5).

### Identification of Genes Related to Immunity

KEGG pathways were reconstructed with KEGG Mapper (41) (version 4.3) based on K numbers identified from the Trinotate blastp annotation. Genes mapping to KEGG pathways within the category “Organismal systems: Immune system” were considered as immune genes. Putative cytokine receptors were identified from KEGG pathways reconstructed based on the *A. queenslandica* blastp annotation (aqu04050 Cytokine receptors). In addition, we screened the reference transcriptome for immune receptors according to the presence of conserved domains (i.e., Pfam domains). In particular, we searched for the presence of TIR domains (PF1582), also in combination with Ig-like domains (PF00047), NACHT domains (PF05729), also in combination with leucine-rich repeat (LRR) domains (PF13516), GPS motifs (PF01825) and seven transmembrane domain (“7TM”), C-type lectin (PF00059) and Scavenger receptor cysteine-rich (SRCR) domains (PF00530 or PF15494). Protein visualization and arrangement of domains was manually checked for identified receptor proteins with SMART (42).

### Quantification of Constitutively Expressed Genes

Constitutively expressed genes were defined as expressed in all 23 samples (i.e., expression level >0 in RSEM expression matrix), regardless of the treatment. TMM (trimmed mean of M values) (43) normalized TPM (transcripts per million) expression was used to explore expression patterns among all sampled sponges (threshold > 10 TMM normalized TPM). Further, the average expression of constitutively expressed genes related to immunity was compared to other genes and differentially expressed genes. Plotting was performed in R with the packages ggplot2 and ComplexHeatmap (version R3.5.1 and 3.6.0) (44, 45).

### Differential Gene Expression Analysis

Gene abundances (Trinity components) were quantified with RSEM (version 1.3.3) for each sample.

Differential gene expression between the control and LPS treatment was analyzed in DESeq2 (within Trinity version 2.8.5 run with R version 3.6.0) For this study, we defined differentially expressed genes (DEGs) as detected by DESeq2 with a FDR p-value < 0.005 and log2-fold change ≥ 2. The DEGs were further assigned to different clusters based on expression pattern similarity (within Trinity version 2.8.5, tree height cut-off 40%) (for more details see **Supplementary Figures 1**, **2**). We observed that in some clusters the expression levels were consistent among the replicates of the same treatment, whereas others showed more variability in the response depending on the individual. Therefore, for further analyses, DEGs were subset into *consistent* and *variable* based on expression clusters (**Supplementary Figures 1**, **2**). Plotting was performed in R with the packages ggplot2 and ComplexHeatmap (version R3.5.1 and 3.6.0) (44, 45).

Gene ontology (GO) enrichment analysis was performed using GOseq for all genes that were found to be significantly up- or down-regulated (46, 47). KEGG pathways were reconstructed with KEGG Mapper (41) (version 4.3) based on K numbers identified from the Trinotate blastp annotation. A protein interaction network analysis was performed in STRING (48) (version 11.0) (default settings with high confidence interaction score 0.700). The network was built on the Clusters of Orthologous Groups of proteins (COG) annotations of the top blastp hit from the *A. queenslandica* proteome. Protein interaction networks were generated with STRING and prettified in Inkscape (version 0.92).

## Results

### *De Novo* Transcriptome Assembly and Annotation

In total, the sequencing approach yielded 2135.98 million paired-end Illumina reads from the 23 sponge samples, which resulted in ~25 million reads per sample after trimming, quality filtering and removal of prokaryotic reads (**Supplementary Table 1**). The generated reference transcriptome showed a high completeness regarding conserved BUSCO genes with 93.5% of the 978 metazoan genes detected while only 4.8% and 1.6% were fragmented or missing, respectively (**Supplementary Table 2**). In total, we identified > 400,000 Trinity components from which 26.7% had an open reading frame (ORF) translating into a protein longer than 100 amino acids. The *de novo* assembly most likely contains several fragments per gene, resulting in an overestimation of total gene number (a common issue in *de novo* assemblies). Thus, one Trinity component does not necessarily correspond to one single gene, and whenever we refer to “genes” in the *H. panicea* transcriptome, we refer to Trinity components or “assembled genes” as identified in the Trinity pipeline. After gene quantification within each sample, on average 80.87 ± 1.63% (average ± standard error) of reads mapped to the reference transcriptome. More details on the *de novo* transcriptome assembly statistics can be found in the supplementary material (**Supplementary Table 2**).

### Diversity of Pattern Recognition Receptors in *H. panicea*

**Figure 1:**
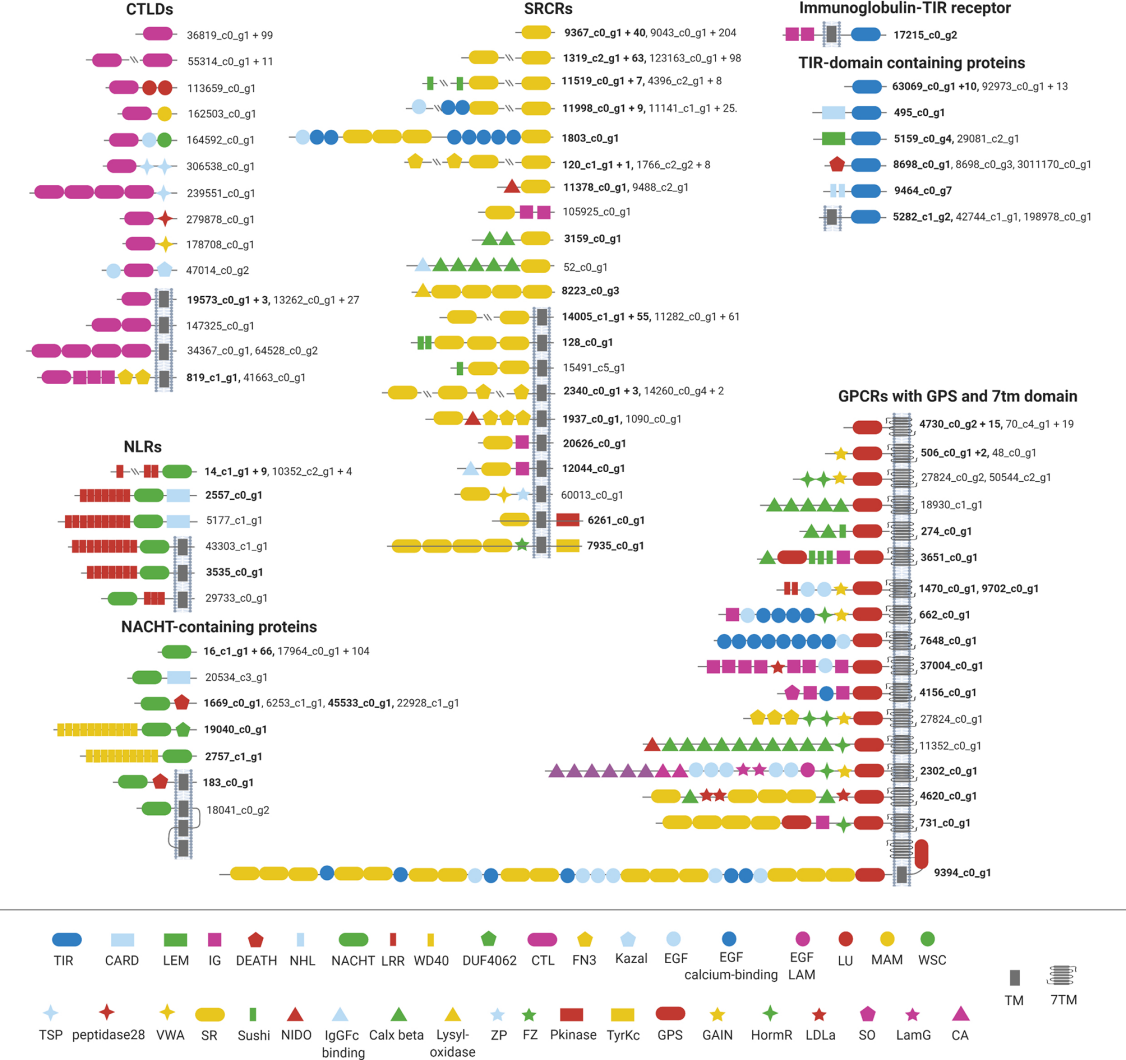
The repertoire of pattern recognition receptors in *Halichondria panicea.* The PRR families GPCRs, SRCRs, CTLD genes, NLRs and TIR-domain receptors were identified based on conserved Pfam domains. Identification numbers of representative transcripts are shown next to protein models, and bold IDs indicate constitutive expression (i.e. present in each analyzed sponge). The number of additional transcripts with the same protein domain architecture is given behind the representative ID. TIR, Toll/interleukine-1 receptor; CARD, caspase recruitment domain; LEM, in nuclear membrane associated proteins; IG, immunoglobulin; NHL, repeat; LRR, leucine-rich repeat; WD40, repeat of 40 amino acids typically terminating in Trp-Asp; DUF4062, conserved domain of unknown function; CTL, C-type lectin; FN3, fibronectin III; Kazal, part of serine protease inhibitors; EGF, epidermal growth factor; EGF-calcium-binding, epidermal growth factor calcium-binding; EGF-LAM, Laminin-type epidermal growth factor-like; LU, Ly-6 antigen/uPA receptor-like; TSP, Thrombospondini domain; peptidase28; VWA, von Willebrand factor; SR, scavenger receptor cysteine-rich; ZP, zona pelludica; FZ, frizzled domain; Pkinase, phosphate kinase; TyrKc, tyrosine-specific kinase; GPS, G-protein-coupled receptor proteolytic site; HormR, present in hormone receptors; LDLa, low density lipoprotein receptor class A repeat; SO, somatomedin B-like; LamG, laminin G; CA, cadherin repeat; TM, transmembrane region; 7TM, 7 helix transmembrane domain. Other domains: DEATH, NACHT, MAM, WSC, Sushi, NIDO, IgGFc-binding, Calx-beta, Lysyl-oxidase, GAIN. The Figure was created with BioRender.com.

To gain an overview of the microbial recognition potential of *H. panicea*, we screened the reference transcriptome for putative PRRs (i.e., non-canonical TLRs, NLRs, CTLD genes, SRCRs and GPCRs) based on conserved protein domains (Pfam annotations) (**Figure 1** and **Supplementary Table 3**). We found one complete non-canonical TLR in *H. panicea* (17215_c0_g2), and as expected (19), no canonical TLRs. We also detected twenty *bona fide* NLRs with a NACHT-domain in combination with LRR domains. Additionally, 181 NACHT-domain containing genes pointed to an even larger variety of NLRs. The reference transcriptome contained a total of 157 CTLD genes and a diverse set of > 600 SRCRs. The characteristic SRCR domains were associated to other domains like Sushi repeats, fibronectin III or epidermal growth factor-like domains like already described for SRCRs in other sponge species (26). 333 GPCR genes contained the distinctive seven transmembrane domain (7tm domain). Due to their extremely diverse domain structure, we focused here on the 57 GPCRs additionally containing a GPCR proteolytic site domain (GPS) (**Figure 1**). Additionally, we detected almost 200 cytokine receptors. Compared to the other receptor classes that can be clearly identified by characteristic, conserved protein domains, cytokine receptors are more heterogeneous. The dominant conserved protein domains among the putative sponge cytokine receptors were tyrosine kinase domains, immunoglobulin domains and fibronectin III, and receptors were classified as receptor tyrosine kinases and TGF-beta receptors. Among all receptor classes we found both membrane-bound (with transmembrane domain), and cytosolic and/or secreted receptors. For the latter, the sequences might be incomplete and the number of membrane-bound receptors thus underestimated.

### PRR Expression Patterns and Constitutive Immune Components

**Figure 2:**
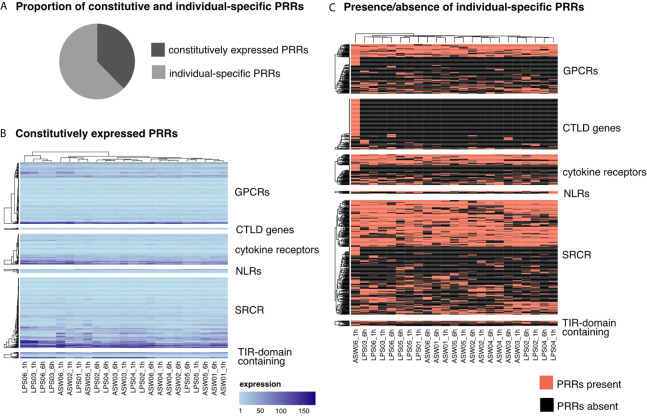
Expression patterns of PRRs. **(A)** 38% of the identified PRRs were constitutively expressed (present in all samples), 62% were expressed individual specific (PRRs present in only some samples). **(B)** Expression levels of constitutively expressed PRRs. Each row represents one gene and each column one sponge sample. The color gradient indicates the expression level (TMM normalized TPM). **(C)** Presence/absence of individual-specific PRRs. Each row represents one gene and each column one sponge sample. Note the clustering of individuals independent of the timepoint (LPS01 was only sampled at 1h and not at 6h). PRR, pattern recognition receptor; GPCR, G-Protein coupled receptor; CTLD, C-type lectin like domain; NLR, nucleotide-binding domain and leucine-rich repeat containing receptors; SRCR, scavenger receptor cysteine-rich; TIR, Toll/interleukin-1 receptor.

Each sponge individual expressed all PRR families but only about 50% of all putative PRR genes detected in the reference transcriptome. Overall, one third of the putative PRRs in *H. panicea* were constitutively expressed, which we defined as expressed in all 23 analyzed samples (**Figure 2A**). The constitutively expressed PRRs covered all PRR families. However, CTLD genes are underrepresented (3% constitutive), whereas GPCRs, *bona fide* NLRs and TIR-domain containing proteins (including the non-canonical TLR) are overrepresented (~50-60% constitutive, respectively). The expression levels of constitutive PRRs were similar across replicate samples (**Figure 2B**). In contrast, we also detected plasticity in PRR expression patterns within the same individual. This was most apparent in one individual expressing an extremely diverse CTLD gene repertoire at 1 h after LPS treatment (125 CTLD genes) compared to 6 h (< 10 CTLD genes) (**Figure 2C**). Nevertheless, the majority of PRRs followed an individual-specific expression pattern (**Figure 2C**).

Then, we examined genes that are constitutively expressed and play a potential role in immunity. Among all 23 analyzed sponge transcriptomes, we identified 28,466 constitutively expressed genes (7% of all genes) (**Supplementary Figure 3A**). From those, we classified a set of 441 genes with a potential function in immunity based on the KEGG mapping results from protein annotations (**Supplementary Table 4**). 61 constitutively expressed genes were related to 19 KEGG orthology (KO) terms relevant for the Toll-like receptor signaling pathway, like NF-κB (K02580) or toll-like receptor 2 (K10159), as well as for the TGF-Beta signaling pathway (e.g. K13375), MAPK signaling (e.g. K04427), apoptosis and TNF signaling like tumor necrosis factor receptor-associated factors (TRAFs) (e.g. K03173), and caspases (e.g. K02187).

The median expression level of constitutively expressed immune genes was ~35% higher than the median of all other constitutive genes (**Supplementary Figure 3A**). We further investigated the subset of constitutive immune genes with higher expression levels (**Supplementary Figure 3B**). As for the PRRs, expression patterns of each of these constitutive immune genes were homogenous across replicates (**Supplementary Figure 3B**). Among these, we highlight those with higher expression levels: two GPCRs (18879_c0_g2, 12713_c0_g1), a cytokine receptor (3010_c1_g1), a nuclear receptor (22428_c0_g1) and a RIG-I-like receptor retinoic acid-inducible gene-I-like receptors) (14596_c0_g1). Further, NF-κB (6780_c0_g1), MyD88 (3294_c2_g1), and several tumor necrosis factor (TNF) receptor-associated factor (TRAF) genes (e.g. 8577_c0_g1, 54071_c0_g1, 196_c1_g1) were also constantly expressed at high levels. The constitutive immune genes with the highest expression were a protease (19175_c0_g1) and two actin binding proteins (91189_c0_g1, 169038_c0_g1).

### Transcriptomic Response to Bacterial LPS Is Highly Individual

We compared the gene expression levels in samples exposed to either LPS or sham (control) at two time points (1 h post treatment and 6 h post treatment) with 6 replicates (but 5 replicates in the LPS treatment at 6 h). The time points were chosen based on results from a similar study in sponges (26). Response to bacterial elicitors is expected to happen and change within a short time frame, where 1 h represents an immediate response, and 6 h a delayed response. Significant differential expression was defined as FDR p-value < 0.005 and log2|FC|≥2.

**Figure 3:**
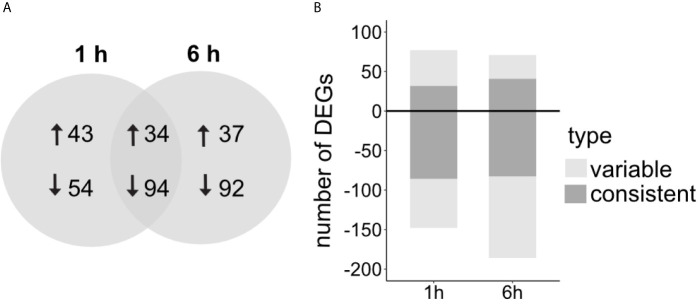
**(A)** Number of DEGs comparing LPS treated versus control sponges and the overlap between the two time points after LPS exposure, 1 h and 6 h after treatment. Arrows indicate up- and downregulation in comparison to control treatment. **(B)** Proportion of *consistent* and *variable* DEGs (with consistent/variable expression patterns across replicate samples, see *Methods*). Total DEGs 1 h: 225, total DEGs 6 h: 257 (genes defined as differentially expressed with FDR p-value < 0.005 and log2|FC|≥2).

Overall, LPS induced more down- than up-regulation of gene expression (**Figure 3A**). The proportion of shared genes between timepoints was larger for the down-regulated genes (~ 40%) than for the up-regulated genes (~ 30%) (**Figure 3A**). DEGs detected at both time points always had the same direction of differential expression and similar expression levels.

**Figure 4:**
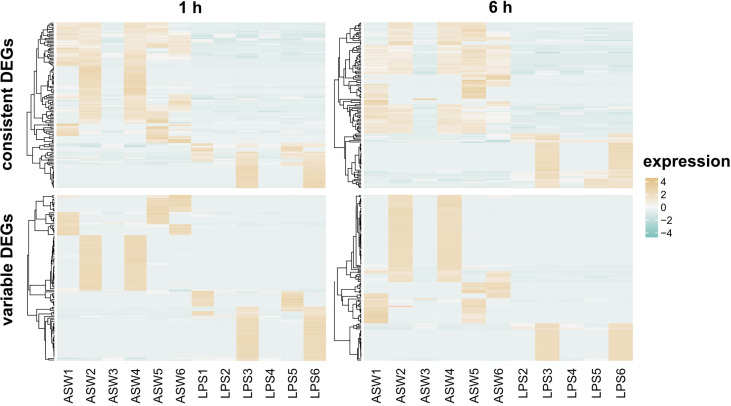
Differentially expressed genes at 1 h and 6 h after LPS exposure. The heatmap shows the TMM-normalized relative expression per DEG (rows) for control and LPS treated samples (columns). DEGs are divided according to expression pattern in *consistent* and *variable* expressed (see *Methods*). Genes were defined as differentially expressed with FDR p-value < 0.005 and log2|FC|≥2.

We realized that not all DEGs show a consistent expression pattern across all replicates (**Figure 4** and **Supplementary Figures 1, 2**). Variability was mainly driven by two individuals in the LPS treatment that seem more responsive to the treatment (**Figure 4**, individual 3 and 6 in LPS treatment). In addition, the control treatment showed high individual variability with individuals 2 and 4 following a similar expression pattern at both timepoints (**Figure 4**). Importantly, some genes identified as differentially expressed were highly expressed in these two control sponges, while they were absent from all other replicates. Because of this individual variability, we clustered DEGs according to their expression patterns (**Figures S1**, **S2**) and, thus, distinguished between *consistent* and *variable* DEGs. DEGs were considered *variable* when they were only regulated in 2 out of the 5/6 replicates per treatment. In contrast, DEGs were considered *consistent* when they showed a similar expression pattern in more than half of the replicates. At 1 h post treatment, 52% of the DEGs were *consistently* regulated, and 48% of the DEGs at the 6 h timepoint (**Figure 3B**).

### Transcriptomic Response to Bacterial LPS Involves Genes Related to Signaling and Recognition

A limiting factor in the functional interpretation of the response of *H. panicea* to LPS is the annotation. No genome for this sponge species is currently available and only about 27% of the DEGs could be annotated based on public databases and conserved protein domains. With additional information retrieved from blastp comparison to the proteome of *Amphimedon queenslandica* (Uniprot UP000007879_444682), we could increase the proportion of annotated DEGs from 27% to ~ 37% (**Supplementary Table 5**).

**Figure 5:**
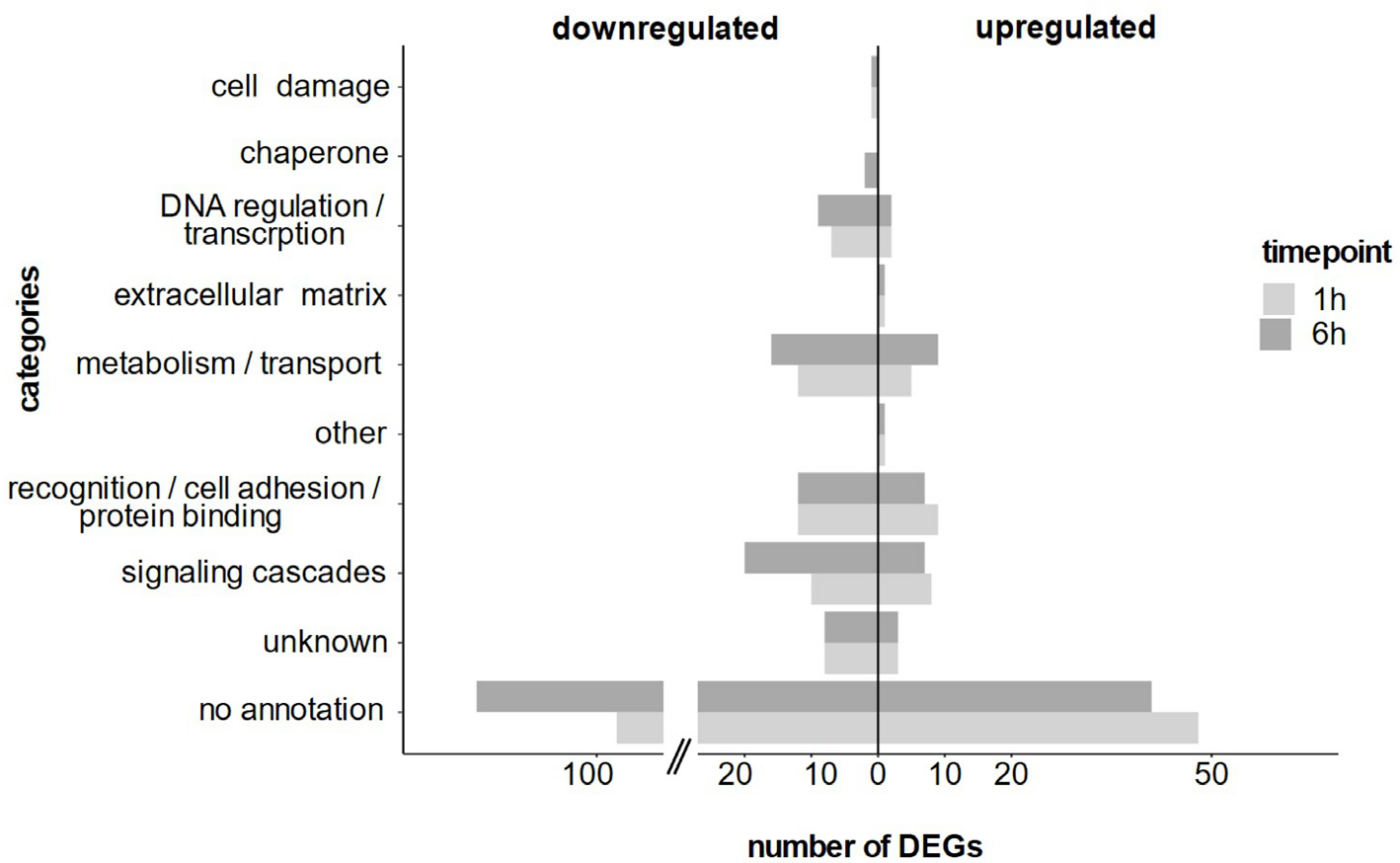
Annotated differentially expressed genes at 1 h and 6 h after LPS exposure separated into functional categories based on their annotation.

At 1 h post LPS treatment most DEGs were functionally related to i) recognition and protein binding, ii) signaling and iii) metabolism and transport (**Figure 5**). Notably, at 6 h post LPS treatment, the same main functional categories were regulated but in varying abundance (**Figure 5**). When considering only the *consistent* DEGs, we detected the same functional categories and temporal differences.

Within genes related to signaling, we found regulation of GTPase activity and GTP binding proteins in response to bacterial LPS. Among the *consistently* up-regulated genes, was a small GTPase with a BTB domain at both timepoints. At 6 h post LPS treatment, two genes involved in semaphorin signaling were also *consistently* upregulated (49): a putative semaphorin receptor (18299_c0_g2) belonging to the plexin group and with a homolog in *A. queenslandica*, and a Sema domain-containing gene that could either function as a semaphorin receptor binding protein or be a receptor itself (8862_c0_g1). Down-regulated genes related to G-Proteins contain several GTPase binding or activating proteins including different septins and small GTPases. We did not detect any G-Protein coupled receptors (GPCRs) among the DEGs.

Several DEGs had typical protein domains involved in recognition, cell adhesion or protein binding: such as ankyrin repeats, Sushi domains, immunoglobulin domains and fibrinogen-like domains. At both timepoints, a gene of the collagen superfamily was *consistently* up-regulated at similar logFC. The signaling response to LPS was also characterized by the regulation of genes annotated as putative tyrosine and serine/threonine kinase activity were differentially expressed at both timepoints, while some of those had homologs in the *A. queenslandica* genome. The majority had *consistent* expression patterns. Genes with kinase activity had different conserved domains including sushi, DEATH or LRR domains. Another *consistent* pattern included the regulation of genes related to ubiquitination, like ubiquitin protein ligases.

Several genes related to the Tumor Necrosis Factor (TNF) signaling pathway were differentially expressed at both timepoints. However, most of these DEGs showed *variable* expression patterns and were only regulated in some of the replicates.

To aid further interpretation of differentially regulated processes after exposure to LPS, we performed a GO-term enrichment analysis and KEGG mapping. The enrichment analysis revealed no significantly enriched GO-terms (FDR corrected p-value < 0.05). *Consistent* DEGs with KEGG annotation were associated to, among others, the KEGG pathways NF-κB signaling (04064), NOD like receptor signaling (04621), IL-17 signaling, TNF signaling (04668) and apoptosis (04210). More specifically, this included genes annotated as TNF receptor-associated factor (TRAF2, TRAF5 and TRAF6), receptor-interacting serine/threonine-protein kinase 1 (RIPK1), and PKR-like endoplasmic reticulum kinases (PERK).

**Figure 6:**
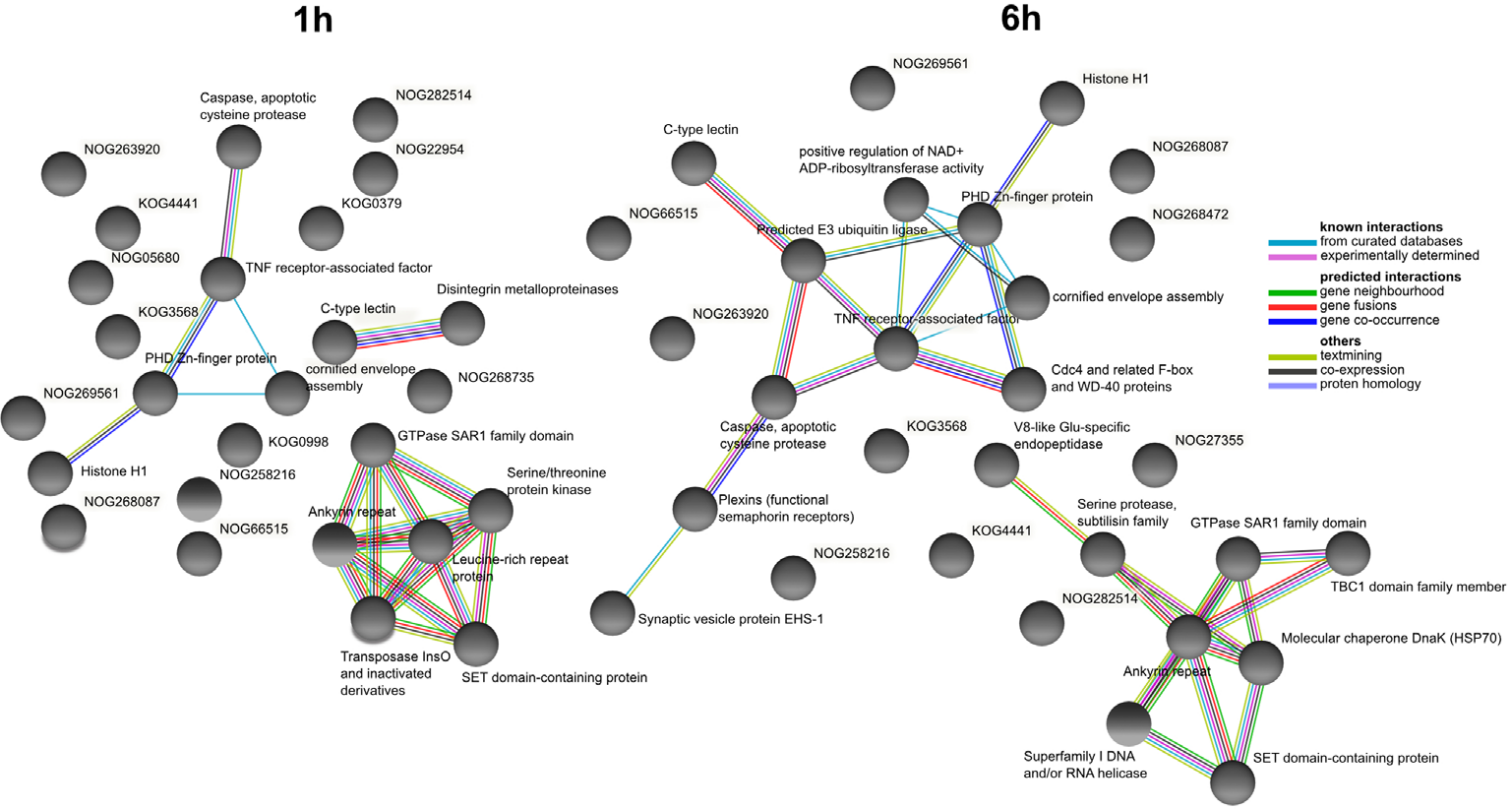
COG association network analysis from DEGs at 1 h and 6 h after LPS exposure identified from closest annotated proteins in *Amphimedon queenslandica*. Created with STRING. Edges represent protein-protein associations coded by color according to the type of evidence for the shown interaction.

At 1h after LPS exposure, the COG association network with the most interactions was centered around leucine-rich repeat proteins and multiple interactions among serine-threonine kinases, GTPases, and ankyrin repeat-containing proteins (**Figure 6**). Distinct networks related to apoptosis (proteases, TNF receptor-associated factors, Zinc-finger proteins) and C-type lectins interacting with proteinases were also identified. At 6 h after LPS exposure, a network showed ankyrin repeat-containing proteins interacting with serine proteases, GTPases, and molecular chaperones. We also observed a network related with apoptosis similar to the one at 1 h, but this time with more connections and centered around TNF receptor-associated factors interacting with caspases, ubiquitin ligases, plexins and WD-40 proteins.

In summary, the main regulated pathways in *H. panicea* upon LPS exposure were related to signaling, recognition, and protein binding, with a large overlap between the timepoints. Despite the variable expression patterns across individuals, all sponges regulated genes in similar functions. The differences lied in the number of regulated genes within each functional category. We did not identify any PRR as differentially expressed. The induced immune response consisted of a network relying predominantly on down-regulation of genes. The transcriptomic response to LPS involved GTP-binding proteins, post-translational regulatory mechanism like ubiquitination, and genes involved in apoptosis.

## Discussion

In this study, we explored the expression patterns of PRRs and characterized the transcriptomic response to LPS challenge in the LMA sponge *H. panicea*. We identified a diverse array of putative PRRs, GPCRs and cytokine receptors. One third of the genes coding for these receptors were expressed at similar levels in all samples (i.e., constitutive PRRs), but each individual also expressed a unique array of PRRs. We further observed high variability between individuals in the genes responding to LPS, although the regulated genes fell under similar functional categories. The differentially expressed genes were predominantly down-regulated and involved genes related to signaling and recognition, such as GTPases, and serine/threonine kinases.

The reference transcriptome assembly of *H. panicea* contained a large diversity of PRRs (**Figure 1**), confirming previous findings based on genomic and transcriptomic information of other sponge species (18, 20, 26, 27). The PRR repertoire in *H. panicea* also agrees with the patterns observed for other LMA sponges, which harbor a more expanded NLR repertoire than HMA sponges (this study, 22, 26, 50). GPCRs and cytokine receptors are not typically considered PRRs, but recent evidence demonstrates their role in recognizing microbial signals (6, 51). Interestingly, the high diversity of cytokine receptors remained hidden with annotation in the standard pipeline (i.e., Trinotate), and was only detected when comparing our dataset to the proteome of the sponge *A. queenslandica*. This lineage-specific annotation suggests a Porifera-specific superfamily of cytokine receptors, as suggested for *A. queenslandica* tyrosine-kinase receptors (52). Most likely, the diversity of cytokine receptors is also large in other sponges and currently underestimated due to the underrepresentation of this phylum in public genomic databases. Further, cytokine receptors do not have a characteristic protein domain in common. Rather, the receptor classes summarized as cytokine receptors are both variable in their architecture and functions [e.g. TGF-β in wounding (53), Eph receptors in cell-cell communication (52)], while their role in immunity is increasingly recognized (54). Cytokine receptors of sponges provide interesting targets for further exploration and promise to harbor novelty.

We evaluated how the PRR diversity and immune-related genes were expressed across samples. Importantly, each individual transcriptome contains representative genes of all PRR families. Moreover, one third of the detected PRR genes were constitutively expressed in all 23 samples (**Figure 2B**). The constitutive immune repertoire of *H. panicea* showed consistent expression levels across samples, suggesting a tight transcriptional regulation and might thus be obligatory for protein function/activity (55, 56). Thus, modulation of these constitutive components could happen *via* post-translational mechanisms like phosphorylation status or binding of adapters. This constitutive and elevated expression is quite intriguing. In plants, overexpression of NLRs negatively affected host health and fitness (reviewed in 57). In the coral *Acropora millepora*, higher disease susceptibility was attributed to elevated constitutive immunity (58). In contrast, we propose that the constitutively expressed genes in *H. panicea* contribute to maintaining microbiome homeostasis and, thus, require sustained expression levels. In LMA sponges, constitutive expression could be higher than in HMA sponges and crucial to maintain a constantly lower microbial load. Jahn et al. recently identified a mechanism for symbionts to silence host immune genes (59). We hypothesize that symbiotic-mediated silencing signals are weaker in the LMA sponges, compared to HMA sponges, allowing the former an elevated constitutive expression. Then, LMA sponges would need to regulate less genes than HMA sponges in response to MAMPs, a pattern observed in two Mediterranean sponges (26). How the density of the microbiome shapes sponge immunity needs to be tested.

The largest proportion of PRR genes showed an individual-specific expression, independent of the timepoint and the treatment (**Figure 2C**). A similar pattern was also found in the purple sea urchin *Strongylocentrotus purpuratus*, where coelomocyte SRCR expression changed on an individual basis (13). The benefit of individual PRR diversity might contribute to the successful response to potential pathogens on the population level by reducing the probability for infection of all individuals simultaneously. For example, in *Drosophila*, individual differences in immune genes are correlated to infection with a gram-negative pathogen (60). Here, differences in the immune repertoire leading to an advantage on the population level (61, 62). This concept has been described in host-pathogen interactions but it may as well apply in the context of proliferation of opportunistic microbes, which seems the real threat for sponges upon environmental stress (17, 63–65).

Variability in PRR diversity occurs at the level of the individual sponge, and thus we argue that the injection with LPS/sham control and a potential associated injury was not the reason for variability. Instead, we propose three possible sources of individual variability. First, variability could result from different genetic backgrounds (e.g. 60). Second, previous exposure and environmental conditions could account for differences between individuals (66). However, in our case all sponges were collected at once from the same location and animals were kept for 2 months in a controlled aquarium system prior to the experiment, further reducing variability. Nevertheless, we cannot discard that previous encounters with microbes have long-term effects or even act over generations, for example, in insects, maternal exposure to pathogens can determine the gene expression of its offspring (67). Third, the potential costs of PRR expression might result in an individual balance between costs and benefit of active immunity in relation, for example, to fitness, as observed in plants (57). We attributed the observed variability mainly to the genotype, as this is rising as a common pattern in other systems, such as for induced immune responses in corals (68).

The individuality observed in the PRR repertoire was also evident in the induced response of *H. panicea* to bacterial LPS, in terms of number of DEGs across individuals. However, the functional categories of the annotated DEGs were consistent and mainly included signaling, recognition, and metabolism (**Figure 5**). We observed the intricate differential expression of multiple GTPases, related to cell-cell interaction *via* G-proteins (69). A particular pathway, *consistently* activated at 6h after LPS exposure, was semaphorin signaling. This pathway is involved in immunity (70) and has its origin in the last common ancestor of choanoflagellates (71). In mice macrophages, semaphorin positively regulated phagocytosis and the inflammatory response after LPS treatment (72). In invertebrates, semaphorins are likely involved in detection and phagocytosis of photosymbionts [in cnidarians (73), in sea slugs (74)]. Sponges rely on phagocytosis for food uptake while depending on differential recognition of their symbionts. Here, semaphorins could also be involved in the discrimination between bacteria. We hypothesize that this function might be conserved from early metazoans to vertebrates.

We see similarities in the transcriptomic response of *H. panicea* to LPS (this study) and the response of Mediterranean sponges *Aplysina aerophoba* (HMA) and *Dysidea avara* (LMA) in response to exposure to LPS and peptidoglycan (26). These similarities are independent of the HMA/LMA dichotomy and the experimental conditions that differed between experiments, and rather reflect a universal response to MAMPs. The three sponge species regulated multiple genes in the category recognition/cell adhesion/protein binding (e.g., immunoglobulin-, leucine-rich repeat- and ankyrin repeat containing genes), and signaling (e.g. TRAFs, protein kinases). GTPases were also regulated in response to MAMPs, but only in *A. aerophoba* we detected regulation of a GPCR. In all three sponges, the gene expression patterns suggest the regulation of apoptosis (e.g. TRAFs, ubiquitination-related genes, proteases). Apoptosis is indeed a common response to microbial elicitation as a mechanism to maintain tissue homeostasis and to restrain infection spread (75, 76). Genes like caspases and ubiquitin ligases, which trigger and regulate apoptosis (77–79), are often activated by LPS in different animal groups like in *C. elegans* (80) and mollusks (81, 82). Apoptosis emerges as a common response to bacterial elicitors in sponges (25, 26, this study), as well as in cnidarians (83, 84) and mollusks (81, 82).

Thus, the gene expression patterns observed in the LMA sponge *Halichondria panicea* show three layers of immune control: (i) a constitutive expression of a subset of PRRs and immune-related genes, (ii) an individual repertoire that expands the constitutive immune array, and (iii) an induced response that acts mainly at the level of signaling cascades (via GTPases) and post-translational regulation of immune components (e.g. *via* ubiquitination and phosphorylation). We propose that the first layer of constitutive genes reflects the low dense *H. panicea* microbiome. We hypothesize that symbiotic-mediated silencing signals (59) are weaker in the LMA sponges, compared to HMA sponges, allowing the former an elevated constitutive expression. The second layer of an individual immune repertoire reflects an individually-determined aspect of immunity. This is an emerging trend in many other organisms and, in fact, this individual variability is well recognized in human medicine and translated increasingly into personalized treatments (85–87). A big question remains: how this individuality may translate into different fitness of marine holobionts upon disturbances (58, 88, 89). The third layer of immunity, i.e. the induced response to LPS, did reflect this individuality. This is a pattern also found in other marine invertebrates like sea urchins and corals (68, 90–92). However, we would like to highlight here the common induced responses, which are differential regulation of signaling by GTPases and post-translational regulation mechanisms, like ubiquitination and protein kinase-mediated phosphorylation. In summary, the discussed layers of immunity would interact with each other in order to determine the specific adequate response. A complex picture of the innate immune control in *H. panicea* emerges, where the different layers act in synergy to maintain a stable microbiome and at the same time mount a flexible response to microbe encounter.

## Conclusion

Here we characterized the patterns of immune gene expression in the emerging LMA sponge model *H. panicea*. We have discovered individuality in both the expressed immune receptor repertoire and the response to the bacterial elicitor LPS. We propose that this individualized immunity may maximize the potential to detect and respond to microbes on the population-level. Our observations further raise the question on how this individualized expressed immune repertoire determines protein function and holobiont fitness in response to a stressor, and whether the amplitude of the induced response affect its costs. The three different layers of immune gene control observed in this study, namely constitutive expression, individual-specific expression, and induced genes, illustrate the complex innate immune gene regulation in *H. panicea*. Most likely this reflects the diverse roles of immunity in sponges interacting with a stable microbiome, seawater bacteria and potential pathogens, and may as well apply to other marine holobionts.

## Data Availability Statement

The raw reads, metadata and transcriptome assembly for this study have been deposited in the European Nucleotide Archive (ENA) at EMBL-EBI under the accession number PRJEB43257 (https://www.ebi.ac.uk/ena/browser/view/PRJEB43257). The full annotation of the de novo transcriptome can be found in **Supplementary Table 6**.

## Author Contributions

LP and LS conceived the idea, planned and conducted the experiment. LS performed molecular laboratory work. SF coordinated and performed RNA-seq at the IKMB Kiel. LS and LP analysed sequencing data and wrote the manuscript. All authors contributed to the article and approved the submitted version.

## Funding

LS and LP were supported by the DFG (CRC1182-B1). LP is also funded by the DFG “Comparative immunogenomics of basal marine metazoans” (IMMUBASE) (project 417981041). This work was supported by the DFG Research Infrastructure NGS_CC (project 407495230) as part of the Next Generation Sequencing Competence Network (project 423957469). NGS analyses were carried out at the Competence Centre for Genomic Analysis (Kiel). LS is supported by the International Max Planck Research School for Evolutionary Biology.

## Conflict of Interest

The authors declare that the research was conducted in the absence of any commercial or financial relationships that could be construed as a potential conflict of interest.
