# Supplementary material for: Individuality in the Immune Repertoire and Induced Response of the Sponge Halichondria panicea
Source: Front Immunol. 2021 Jun 16;12:689051. doi: 10.3389/fimmu.2021.689051 (PMC8242945; doi:10.3389/fimmu.2021.689051)
Supplement: Supplementary file 1 [file Image_1.pdf]

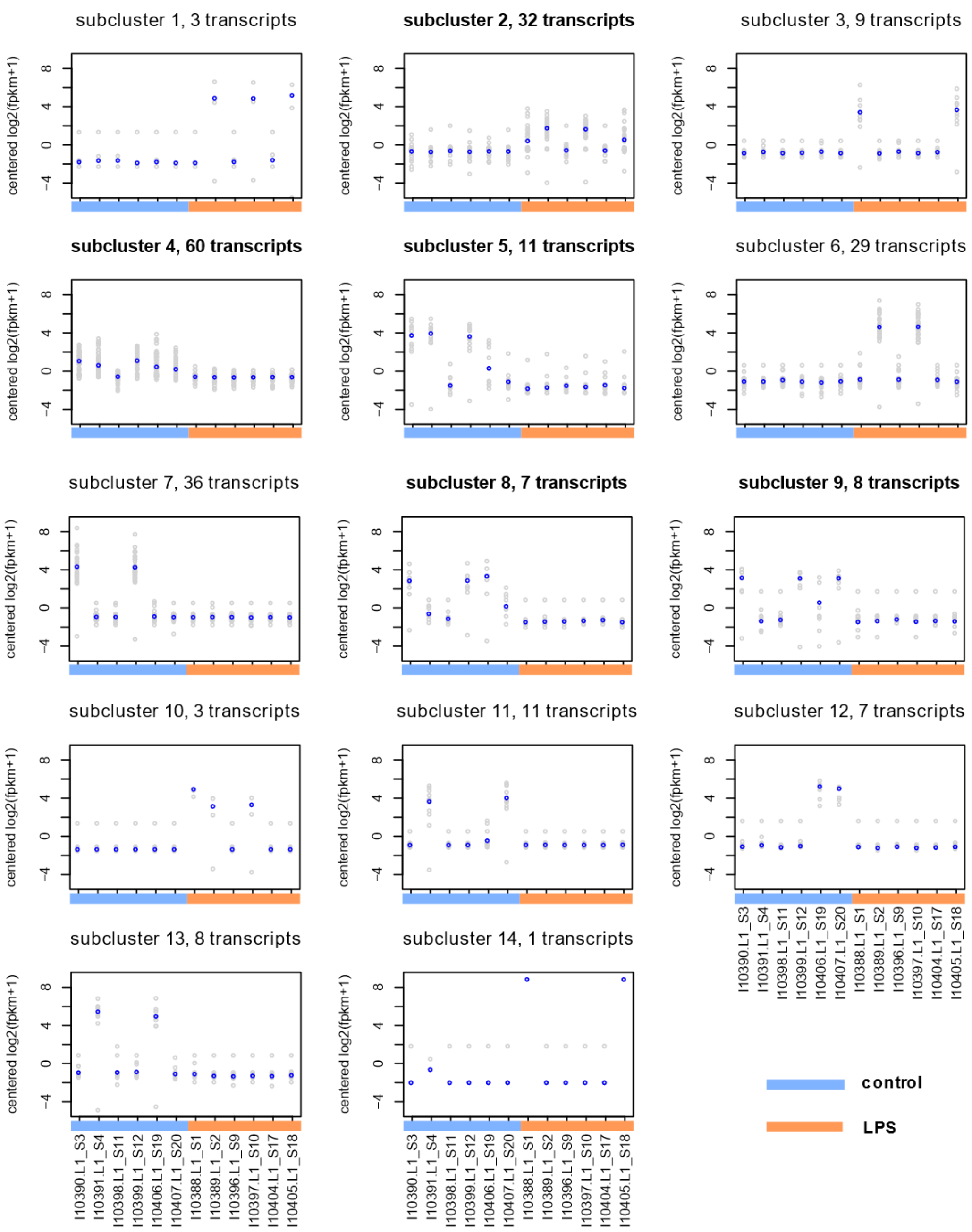

Supplementary Figure 1: Subclustering of differentially expressed transcripts at 1 h post LPS treatment. Transcript sets with related expression patterns were built based on the hierarchically clustered transcript tree (cut-off: 40 % tree height). Mean-centered expression is shown for each sample. Subsets were considered *consistently* expressed when more than half of the replicates showed up/down regulation and are indicated in bold letters (subcluster 2, 4, 5, 8 and 9 with 118 transcripts in total).
