# Supplementary material for: Individuality in the Immune Repertoire and Induced Response of the Sponge Halichondria panicea
Source: Front Immunol. 2021 Jun 16;12:689051. doi: 10.3389/fimmu.2021.689051 (PMC8242945; doi:10.3389/fimmu.2021.689051)
Supplement: Supplementary file 2 [file Image_2.pdf]

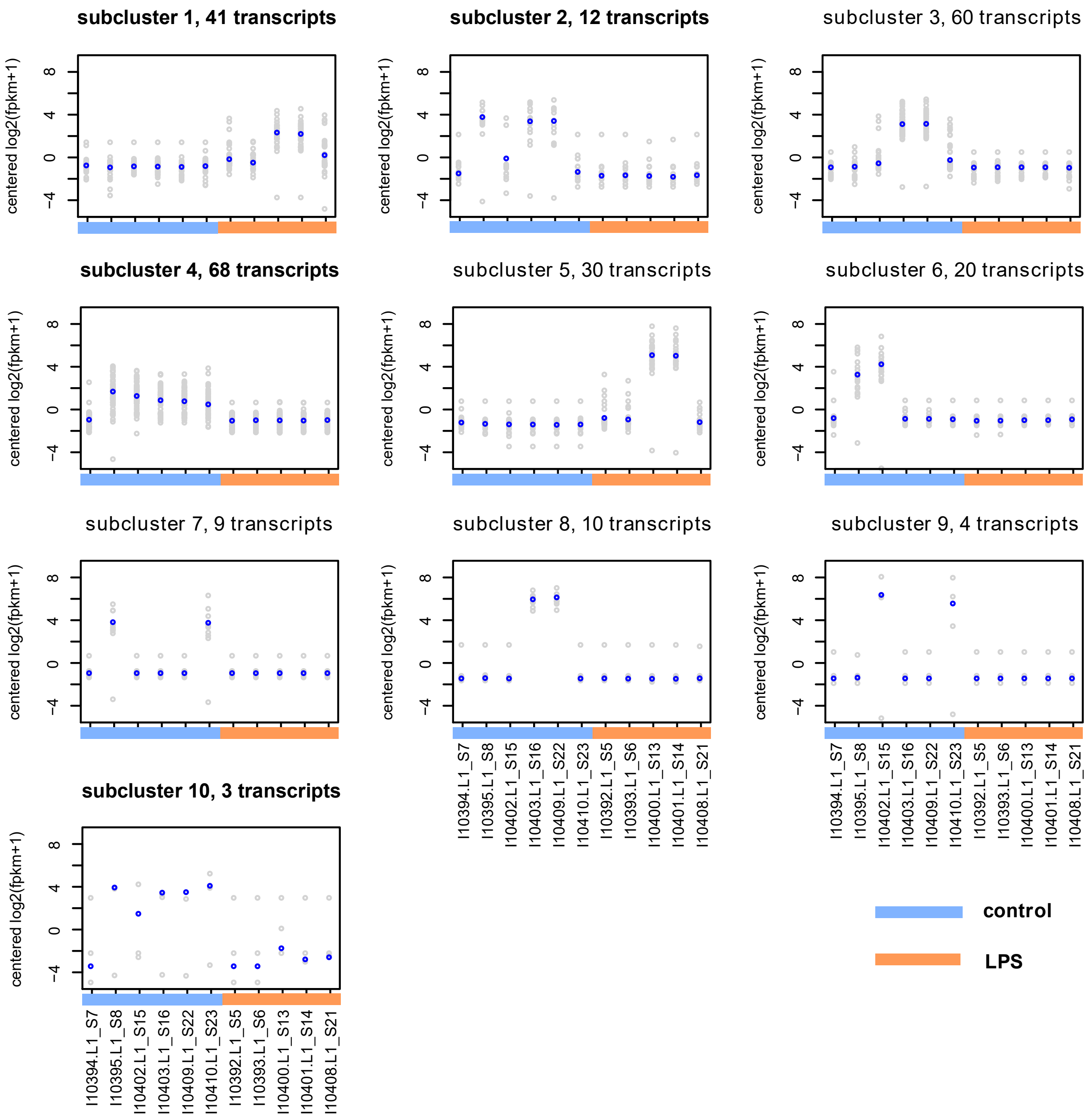

Supplementary Figure 2: Subclustering of differentially expressed transcripts at 6 h post LPS treatment. Transcript sets with related expression patterns were built based on the hierarchically clustered transcript tree (cut-off: 40 % tree height). Mean-centered expression is shown for each sample. Subsets were considered *consistently* expressed when more than half of the replicates showed up/down regulation and are indicated in bold letters (subcluster 1, 2, 4 and 10 with 124 transcripts in total).
