# Supplementary material for: Individuality in the Immune Repertoire and Induced Response of the Sponge Halichondria panicea
Source: Front Immunol. 2021 Jun 16;12:689051. doi: 10.3389/fimmu.2021.689051 (PMC8242945; doi:10.3389/fimmu.2021.689051)
Supplement: Supplementary file 3 [file Image_3.pdf]

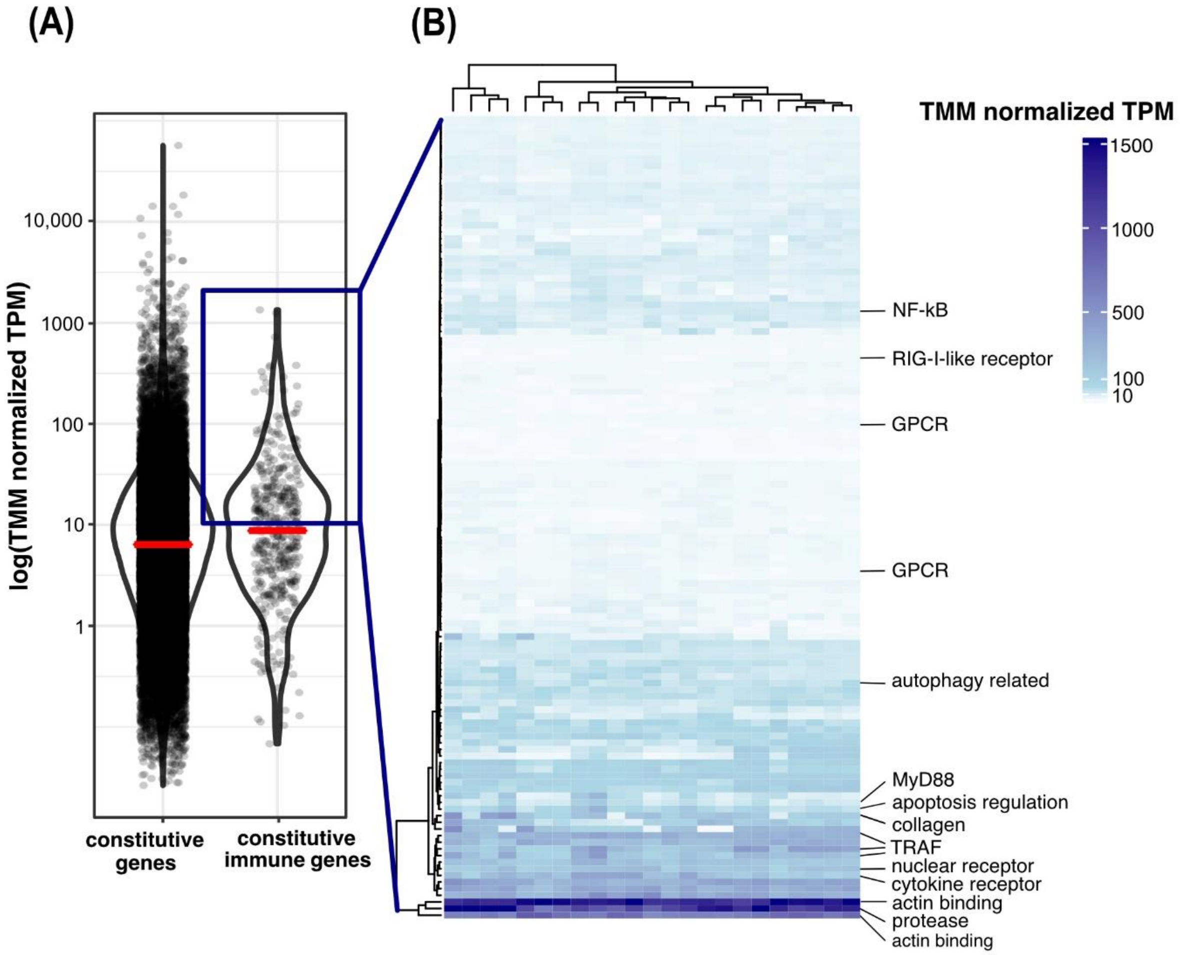

Supplementary Figure 3: Expression levels of constitutive genes (i.e. expressed in all samples) related to immunity. A: Expression levels (TMM normalized TPM) of constitutive genes in the reference transcriptome and constitutive genes related to immunity. Each dot represents the average expression level in the 23 analyzed sponges, the red bars represent the median expression per category (constitutive genes: 6.39 TMM normalized TPM, constitutive immune genes: 8.76 TMM normalized TPM). B: Heatmap of highly expressed immune genes (TMM normalized TPM > 10). NF-κB = nuclear factor kappa of activated B-cells, GPCR = G-Protein coupled receptor, MyD88 = myeloid differentiation primary response 88, RIG-I-like receptor = retinoic acid-inducible gene-I-like receptors, TRAF= tumor necrosis factor (TNF) receptor-associated factor.
